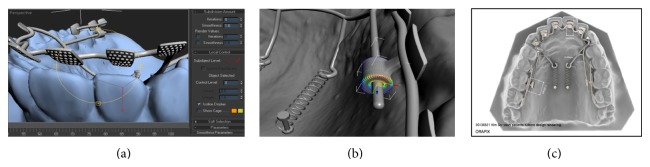# Accelerated Tooth Movement and Temporary Skeletal Anchorage Devices (TSADs)

**DOI:** 10.1155/2014/384380

**Published:** 2014-12-18

**Authors:** Seong-Hun Kim, Shin-Jae Lee, Gerald Nelson, Ki Beom Kim

**Affiliations:** ^1^Department of Orthodontics, School of Dentistry, Kyung Hee University, Seoul 130-701, Republic of Korea; ^2^Department of Orthodontics, School of Dentistry and Dental Research Institute, Seoul National University, Seoul 110-749, Republic of Korea; ^3^Division of Orthodontics, Department of Orofacial Science, University of California, San Francisco, CA 94143, USA; ^4^Department of Orthodontics, Center for Advanced Dental Education, Saint Louis University, 3320 Rutger Street, Saint Louis, MO 63104, USA

Accelerated tooth movement (ATM) has been one of the vital treatment considerations in orthodontics. Currently, accelerated tooth movement is not limited in orthodontics only, but it extends into other parts of dentistry, such as periodontics, oral surgery, and prosthodontics. More and more, the dental treatments are approached interdisciplinary to achieve the best treatment outcomes and long-term prognosis in patients. There are increasing interests in defining the biologic changes to enhance the tooth movement and numerous* in vitro* and* in vivo* experimental studies contributed in understanding the modulators to enhance faster tooth movement to apply in mechanics. In accelerating the tooth movement, temporary skeletal anchorage devices (TSADs) have become one of the clinical modalities that gained so much recognition. TSADs can aid in targeting tooth movement control by providing skeletal anchorage without depending on adjacent teeth. In clinical practice, the combination of ATM and TSADs together can further advance treatment efficiency and efficacy of patients achieving treatment goals. The stability of TSADs is essential to fulfill the purpose of the treatment. This special issue delivers original research on the different aspects of TSADs' stability and also provides clinical research on new treatment method for ATM utilizing TSADs. N. Kaipatur et al.'s study was to develop an FE model of a TSAD in the rat maxilla to estimate the stress distribution in the surrounding cortical bone and the TSAD stability at different force levels followed by* in vivo* validation using a rodent model of orthodontic tooth movement. The strength of this article stems from the fact that there is no study published to date that used microimplants as TSAD for direct anchorage to facilitate tooth movement. Most of the studies that published on tooth movement used incisors as anchorage with iatrogenic and deleterious effects and concern for animal welfare. The significant amount of tooth movement they found would not have been possible without TSAD stability and resultant constant force levels although 6.7 microns/day distal drift and cranial growth could have had a minor influence on implant stability measurement.

The stability of TSADs in healthy patients has been proved through numerous researches and journals. However, it would be meaningful to figure out factors that influence the stability of TSADs in systemic diseased patients. Diabetes mellitus affects bone healing and so it poses risk in stability of TSADs. J.-B. Park et al. and N.-H. Oh et al. made various attempts to improve the success rate of TSADs in DM patients. J.-B. Park et al.'s study aimed to evaluate effects of type 1 diabetes mellitus and mini-implant placement method on the primary stability of mini-implants by comparing mechanical stability and microstructural/histological differences. Through their animal study, type 1 diabetes mellitus and placement method of mini-implant did not affect primary stability of mini-implants.

Study of N.-H. Oh et al. was to investigate effects of surface treatment of mini-implants in diabetes-induced rabbits by comparing osseointegration around mini-implants. In surface-treated mini-implants, maximum removal torque was higher in both diabetic and control groups. Type 1 diabetes mellitus and surface treatment method of mini-implant affected primary stability of mini-implants. In addition, the use of orthodontic mini-implants in a diabetic patient is likely to show results similar to that of the healthy patient. Further study is necessary; nevertheless from the studies by J.-B. Park et al. and N.-H. Oh et al., it has been confirmed that the surface treatment of TSADs and the implant method influence the stability of TSADs. When loosening and failure were experienced during TSADs usage, they were often replanted via recycling in a patient. Study by S. Estelita et al. provides another solution about the consequences of mechanical stimulation occurring during recycling of TSADs on mechanical stability of mini-implants.

They evaluated the influence of recycling process on the torsional strength of mini-implants. The recycling protocols did not influence torsional strength of bone screws even when sandblast cleaning produced an abrasive mechanical stripping of the screw surface, but the structural loss was not sufficient to significantly influence the fracture torque. The stability of TSADs with minimum number of TSADs for enabling the detailed 3-dimensional tooth movement has been suggested by 3D CAD/CAM clinical study of S.-Y. Kwon et al. In their study, custom lingual orthodontic appliances named kinematics of lingual bar on nonparalleling technique (KILBON) were virtually designed by merging 3D model images with lateral and posterior-anterior cephalograms (Figure 1). This report describes CAD/CAM fabrication of the complex anteroposterior lingual bonded retraction appliance for intrusive retraction of the maxillary anterior dentition.

In conclusion, TSADs have tremendously broadened the orthodontic treatment scope and impacted on reducing the need for surgical treatment. Application of TSADs for the ATM and the stability of TSADs within alveolar bone are critical; therefore, the original articles in this special issue on the methods of TSADs implantation and surface finish in terms of stability would pose significant role in future of contemporary orthodontics. In addition, recent advancement of 3D CAD/CAM imaging would permit least number of TSADs for the maximum treatment effect accurately. There are anatomic limitations in placement of TSADs and orthodontic tooth movement and also challenges in the improvement of medical image for detailed CAD/CAM appliance fabrications; nonetheless, the progress of science and the further researches would overcome these obstacles.

*Seong-Hun Kim* *Seong-Hun Kim* *Shin-Jae Lee* *Shin-Jae Lee* *Gerald Nelson* *Gerald Nelson* *Ki Beom Kim* *Ki Beom Kim*

## Figures and Tables

**Figure 1:**